# Mining Label Data: Assessing the Presence of *ortho*-Phthalates in Pharmaceuticals and Dietary Supplements

**DOI:** 10.1289/ehp.120-a123b

**Published:** 2012-03-01

**Authors:** Tanya Tillett

**Affiliations:** Tanya Tillett, MA, of Durham, NC, is a staff writer/editor for *EHP*. She has been on the *EHP* staff since 2000 and has represented the journal at national and international conferences.

Plasticizers known as phthalates are used in many medical and household products. They also are used in the coatings on timed-release pharmaceuticals and dietary supplements, and high levels of phthalate metabolites have been found in subjects who take such products regularly. In a new study, investigators examined available data to determine the extent and scope of *ortho*-phthalate use in pharmaceutical and dietary supplement products marketed in the United States and Canada since 1995 [*EHP* 120(3):379–384; Kelley et al.].

Past studies in animals have shown that some *ortho*-phthalates such as di(2-ethylhexyl) phthalate (DEHP) and di-*n*-butyl phthalate (DBP) exhibit reproductive and developmental toxicity, although no such effects were seen for diethyl phthalate (DEP). Limited data suggest that all 3 compounds may adversely affect male reproductive health in humans.

*ortho*-Phthalates are used extensively as excipients (inactive ingredients) in modified-release medications taken by mouth. These include drugs delivered through controlled release, delayed release, or targeted release systems. Depending on the product, the phthalate coating may protect ingredients from being degraded prematurely by stomach acid, reduce stomach irritation, minimize aftertaste, or make the product easier to swallow.

In the current study, investigators searched a variety of print and electronic information sources to identify brand-name and generic medications and dietary supplements sold in the United States and Canada that contained phthalates as excipients. They identified more than 100 such products, including 50 prescription medications, 40 over-the-counter products, and 26 dietary supplements. Most of these contained DEP; 9 products contained DBP, and 1 contained both DEP and DBP.

The researchers were unable to produce a truly comprehensive list because of the lack of a centralized data retrieval source, reliance on pharmaceutical companies to accurately disclose ingredient lists for specific products, and proprietary considerations for particular formulations. However, the study does give researchers an extensive, systematically catalogued list to refer to when conducting future studies aimed at analyzing the potential risks and human health effects associated with exposure to phthalates. The authors recommend that future studies consider the amount of phthalate that is used in various forms of medicinal products in order to estimate *ortho*-phthalate exposure from these sources.

